# Antifungal effect of triclosan on *Aspergillus fumigatus*: quorum quenching role as a single agent and synergy with liposomal amphotericin-B

**DOI:** 10.1007/s11274-022-03325-1

**Published:** 2022-06-20

**Authors:** Roya Tamimi, Godfrey Kyazze, Tajalli Keshavarz

**Affiliations:** School of Life Sciences, College of Liberal Arts and Sciences, University of Westminster, London, W1W 6UW UK

**Keywords:** Biofilm, Combination treatment, Conidia, Flow cytometry, Quorum quenching

## Abstract

The purpose of this research was to determine *Aspergillus fumigatus* conidial viability and its biofilm formation upon treatment with triclosan and amphotericin-B loaded liposomes. *A. fumigatus* was treated with the antimicrobials, triclosan and liposomal amphotericin-B (L-AMB), in single and combined supplementation. To quantify the cells’ viability upon treatments, resazurin-based viability assay was performed. Confocal laser scanning microscopy was done by applying FUN-1 stain to screen the role of the agents on extracellular polymeric substances. Total *A. fumigatus* biomass upon treatments was estimated by using crystal violet-based assay. To study the agents’ effect on the conidial viability, flow cytometry analysis was performed. Expression levels of *A. fumigatus* genes encoding cell wall proteins, α-(1,3)-glucans and galactosaminogalactan were analysed by real-time polymerase chain reaction assay. A synergistic interaction occurred between triclosan and L-AMB when they were added sequentially (triclosan + L-AMB) at their sub-minimum inhibitory concentrations, the triclosan and L-AMB MICs were dropped to 0.6 and 0.2 mg/L, respectively, from 2 to 1 mg/L. Besides, L-AMB and triclosan contributed to the down-regulation of α-(1,3)-glucan and galactosaminogalactan in *A. fumigatus* conidia and resulted in less conidia aggregation and mycelia adhesion to the biotic/abiotic surfaces; *A. fumigatus* conidia-became hydrophilic upon treatment, as a result of rodlet layer being masked by a hydrophilic layer or modified by the ionic strength of the rodlet layer. In *A. fumigatus*, the potential mechanisms of action for L-AMB might be through killing the cells and for triclosan through interrupting the cells’ development as a consequence of quorum quenching.

**Supplementary Information:**

The online version contains supplementary material available at 10.1007/s11274-022-03325-1.

## Introduction

Biofilm formation is implicated in the pathogenesis of localized as well as invasive *Aspergillus fumigatus* (*A. fumigatus*) diseases (Beauvais et al. 2007; Mowat et al. 2007; Chandrasekar and Manavathu 2008). Aspergilloma, a localized infection, and invasive pulmonary aspergillosis (IPA) have been shown to form biofilms containing highly agglutinated hyphae of *A. fumigatus* encased in extracellular polymeric substances (EPS) (Mowat et al. 2008; Loussert et al. 2010). Amphotericin B (AMB) is an antifungal agent and as a polyene antibiotic, it binds to ergosterol in the fungal cell membrane and through a series of steps destroys the cells. However, as biofilms are highly resistant to antimicrobials, *A. fumigatus* colonies encased in EPS are more resilient to AMB (Beauvais et al. 2007). It has been demonstrated that during early to intermediate phases of biofilm development, EPS are produced close to the fungi maturity stage (Ramage et al. 2011). Microbial cells facilitate, through EPS production, the attachment of other pathogens by providing more diverse adhesion sites on biotic and abiotic surfaces. EPS holds the biofilm together, leading to an irreversible cell attachment to the surfaces (Flemming and Wingender 2010). Thus, EPS formation and degradation can be used as a marker for the antifungal activity of an agent.

As a major constituent in *A. fumigatus* biofilm EPS, Galactosaminogalactan (GAGs) plays a critical role in maintenance of the EPS (Loussert et al. 2010). Activity of *sph3*, which encodes a protein belonging to the spherulin 4 family is required for the synthesis of functional GAG (Bamford et al. 2015). Strains deficient in GAG fail to produce EPS and are unable to form adherent biofilms on plastics or host cells in vitro (Gravelat et al. 2013). GAG-mediated adherence is largely a consequence of charge interactions between the polycationic polysaccharides of the fungus cell wall and negatively charged surfaces (Lee and Sheppard 2016). Cell wall α-(1,3)-glucans play an essential role in conidia (asexual spores) aggregation (Fontaine et al. 2010; Henry et al. 2011). In particular, three α(1–3)-glucan synthase genes, *ags1*, *ags2*, and *ags3*, have been found to be responsible for cell wall α(1–3)-glucan biosynthesis. Among these genes, only *ags3* is involved in virulence (Beauvais et al. 2005; Maubon et al. 2006). Taken together, α-(1,3)-glucan and GAG down-regulation might result in less conidia aggregation and mycelia attachment to the biotic/abiotic surfaces, respectively.

Quorum sensing (QS) signals contribute directly to microbes’ pathogenesis due to the production of virulence determinants such as toxins, proteases, and immune-evasive factors (Scoffone et al. 2019). Quorum quenchers as biostatic agents affect QS pathways and hence biofilm formation. Biostatic agents are increasingly favoured over microbicidal agents as they do not appear to trigger resistance in microbes (Fleitas Martínez et al. 2019; Lu et al. 2022). Targeting components of microbes that are responsible for pathogenesis, makes quorum quenchers better candidates compared to the agents with growth-inhibitory effects.

Triclosan (C_12_H_7_Cl_3_O_2_) is an antiseptic diphenyl ether derivative used in toothpaste, mouthwash, surgical soaps, and cosmetics (Weatherly and Gosse 2017). It disrupts biofilm formation in some bacteria, yeasts, and dermatophytes through blocking the biosynthesis of amino acids and fatty acids (Hoang and Schweizer 1999; Jones et al. 2000). Features such as nuclear chromatin condensation, substantial intracellular vacuolation and mitochondrial swelling were shown by triclosan-treated *Cryptococcus neoformans* (*C. neoformans*) suggesting that triclosan induced apoptosis-like cell death (Movahed et al. 2016). However, the efficacy of triclosan against filamentous fungi has not been studied so far.

Synergism has been identified between triclosan and two principal drugs, AMB and fluconazole against *C. neoformans* (Movahed et al. 2016). The study applied microdilution checkerboard assay and viewed cell morphology under transmission electron microscope. In Tobudic et al. (2010) synergism was demonstrated by the combination of AMB/Posaconazole with improved effectiveness against *C. albicans* biofilms. Mesquita et al. (2013) used flow cytometry to investigate the effect of disinfection therapy on the survival, development, and metabolic activity of gamma-radiated fungal spores (*Penicillium chrysogenum*, *Aspergillus nidulans*, and *Aspergillus niger*).

Resistance of *A. fumigatus* towards AMB, and the side effects of single use triclosan as a synthetic antimicrobial agent on the environment and human health are the concerns which can be overcome through combination therapy.

In this study we used liposomal AMB (L-AMB), which compared to the conventional AMB deoxycholate, has the same antifungal activity following its incorporation into a liposome bilayer, while its toxicity against human organs is significantly lower (Stone et al. 2016). We also investigated the potential quorum quenching role of triclosan against *A. fumigatus* and compared it to the efficacy of L-AMB. Furthermore, we studied the combination of triclosan and L-AMB against *A. fumigatus* viability.

Persister cells (also called drug-tolerant cells), which include approximately ≤ 1% of the overall biofilm population, are considered as a possible reason for drug resistance. Persister cells neither grow nor die in the presence of microbicidal agents (Keren et al. 2004; Lewis 2007). Triggering persister cells by an antimicrobial agent, and hence making them vulnerable against another agent, is considered as a strategy for a combination treatment. In this context, we investigated the synergy between triclosan and L-AMB through their effect on triclosan triggered persister cells.

## Materials and methods

### The fungus and its maintenance

*A. fumigatus* ATCC46645, was obtained from the Culture Collection of the University of Westminster, London, UK. Stock cultures of *A. fumigatus* maintained on potato dextrose agar (PDA) (Merck, Dorset, UK,) medium were propagated in potato dextrose broth (PDB) (Fisher Scientific, Loughborough, UK) or RPMI-1640 (Merck, Dorset, UK).

### Culture and treatment of *A. fumigatus* for resazurin-based viability assessment

Minimum inhibitory concentration (MIC) is defined as the lowest concentration of an antimicrobial that will inhibit the visible growth of a microorganism after overnight incubation (Andrews 2001). Different doses of the antimicrobial agents, triclosan (Sigma-Aldrich, Dorset, UK) and L-AMB (Thermo Fisher Scientific, Leicestershire, UK) were applied to *A. fumigatus* at t_0_ and after 24 (represents early biofilm structure) and 48 (represents mature biofilm structure) hrs*.* To measure fungus viability, resazurin dye solution (Merck, Dorset, UK) was added to the culture (10% v/v) in 96-well plates. The plates were then incubated at 37 °C on a shaker at 50 rpm for 50 min and the absorbance was measured spectrophotometrically at a wavelength of 590 nm. Single and combination treatments of *A. fumigatus* with antimicrobial agents, triclosan and L-AMB, were performed. For that, conidia and sessile (biofilm) cell viabilities were measured by In vitro toxicology assay kit (Merck, Dorset, UK) in 96-well tissue culture plates (Corning Inc., Corning, NY).

### Treatment of *A. fumigatus* with triclosan and L-AMB

For single treatment, in a 96-well microplate, RPMI-1640 medium (200 µL) containing the agent’s effective dose and inoculum (10^6^ conidia/mL) was added per well. After incubation at 37 °C for 24 h, the cells’ metabolic activity was determined through resazurin assay.

Triclosan-L-AMB interactions were evaluated using the checkerboard assay. Checkerboards were prepared by using serial dilutions of L-AMB and triclosan. Triclosan and L-AMB dilutions were prepared as recommended in the EUCAST (European Committee on Antimicrobial Susceptibility Testing) protocol to give final drug concentrations of 0.3, 0.6 and 1.2 mg/L and 0.1, 0.2 and 0.4 mg/L for triclosan and L-AMB, respectively, in 100 µL of double-strength RPMI medium containing 10^6^ conidia/mL fresh spore suspension. Subsequently, 100 µL of RPMI medium containing fresh spore suspension (10^6^ conidia/mL) were added to the wells, resulting in concentrations of 0.15, 0.3 and 0.6 mg/L and 0.05, 0.1 and 0.2 mg/L for triclosan and L-AMB, respectively. For both simultaneous and continuous combination treatment strategies, viability was determined by using resazurin assay after 48 h treatment at 37 °C. The OD at 570 nm wavelength was determined with a spectrophotometer (Jenway 6300 visible; Camlab Limited, UK). Fractional inhibitory concentration index (FICI) was calculated as follow:

1 $$FICI=(MICAcomA,B \div MICagentA)+(MICBcomA,B \div MICagentB)$$

According to the above equation, FICI ≤ 0.5 indicates synergy, FICI > 4 indicates antagonism whereas 0.5 < FICI < 4 suggests no interaction. To study the synergistic assay, Compusyn software (ComboSyn, Inc.) was used. The resulting CI theorem of Chou-Talalay offers quantitative definition for additive effect (CI = 1), synergism (CI < 1), and antagonism (CI > 1) in drug combinations (Chou 2010).

### Evaluation of the EPS and biofilm depth by using confocal laser scanning microscopy

Leica SP2 LSCM (CLSM, Carl Zeiss, Jena, Germany) was used to examine the fluorescent filamentous biomass and hence the biofilm and its thickness. *A. fumigatus* biofilm formation on glass slides after 48 h of incubation at 37 °C were analysed using FUN-1-based confocal laser scanning microscopy (CLSM). Microscopic visualization and image acquisition of biofilms were conducted using an upright scanning Leica confocal microscope equipped with an argon/krypton laser and detectors, and filter sets for monitoring of green (excitation 480 nm, emission 517 nm) and red (excitation 633 nm, emission 676 nm). The biofilms on the surfaces were washed three times in sterile PBS and stained using a fluorescent stain, FUN-1 (Molecular Probes, Life Tech) prepared according to the manufacturer’s instructions. For biofilm visualization, FUN-1 (1 μL) from a 10 mM stock was mixed in 1 mL of PBS. Three drops of the mixture were added on the top of the biofilm, which was then mounted on a coverslip. The slides were incubated for 45 min at 37 °C in the dark. The biofilm, formed as explained above, was washed again with PBS and mounted on a slide. An excitation wavelength of 488 nm using an argon ion laser at a magnification of × 200 was used to examine the biofilms. Horizontal (xy) view of reconstructed 3-dimensional images of FUN1-stained biofilms was applied to capture green fluorescence.

Thickness of the biofilm was observed in the side view of the reconstruction. Sections on the xy plane were taken at 1 μm intervals along the z-axis of the sections taken parallel to the x–y plane to determine the depth of the biofilms. The CLSM analysis of the biofilms was used to measure and to compare the “means” of the thickness of the triclosan-treated and the combination-treated test groups. Three-dimensional images were assembled using Leica Confocal Software.

### Biofilm quantification by crystal violet assay

The effect of the agents (triclosan and L-AMB at their MICs) on the mature biofilm (36 h of incubation) was estimated using the crystal violet (CV) assay. The assay stains both live and dead cells as well as some components present in the biofilm matrix, thus it is well suited to quantify total biofilm biomass. To determine the ability to form biofilms, an inoculum (10^6^ conidia/mL) was added to 200 µL PDB medium in a 96-well polystyrene microtiter plate, which was incubated, without agitation, at 37 °C for 36 h. Subsequently, the medium was aspirated and non-adherent cells removed by thoroughly washing the formed biofilm three times with PBS. After fixation with glutaraldehyde (70% in H_2_O; Sigma-Aldrich, UK) for 20 min and air-drying, the biofilms were stained with 200 µL, 0.5% w/v CV (Merck, Dorset, UK) for 15 min, followed by rinsing with sterile PBS, and de-staining with 96% v/v ethanol (VWR, Brooklyn, NY). The absorbance of the CV-stained biofilm in the treated and untreated control groups was measured at 590 nm using a spectrophotometer. Percentage biofilm biomass in the treated samples was calculated using the following equation:

2 $$A\,590\,of\,the\,test \div A\,590\,of\,untreated\,control) \times 100$$

where A_590_ is the absorbance of the CV-stained biofilm matrix at 590 nm.

### Viability assay of the conidia by using flow cytometry

Propidium iodide (PI) is a positively charged, membrane-impermeable fluorochrome that can only pass through the membranes of stressed, injured, or dead cells. It has been already shown that flow cytometry can be applied on conidia cells to assay their viabilities (Balajee and Marr 2002; Mesquita et al. 2013; Vanhauteghem et al. 2017).

To assess conidia viability, protocol defined by Mesquita et al. (2013) was adopted and filtered sterile water was used instead of culture media. Flow cytometry tubes (3.5 mL) using control tubes and experimental tubes were prepared (1 mL of deionised water + 1 mL of the conidia suspension). Subsequently, 100 µL of each treated and untreated control group was added to a flow cytometry tube with 50 µL of PI (Abcam, Cambridge, UK) stock solution (1 mg/mL), and 1.85 mL deionised water. After 10 min of staining with PI, tubes were then mixed and analysed using the cytometer (NovoCyte Benchtop, ACEA Biosciences, UK). PI is excited at 488–523 nm. It fluoresces orange-red and can be detected using a 562–588 nm band pass filter. Conidial viability was defined by the FSC (forward scatter) and SSC (side scatter) characteristics and the mean fluorescence intensity (MFI) of PI in the treated and untreated conidia. Within scatter parameters, the pulse height vs pulse width plots (FSC vs SSC) are used to identify cells of interest based on size and granularity and to isolate single cells passing through the cytometer, thereby removing any non-single cells (doublets, clumps and debris) (Rowley 2012).

### Real-time-PCR assay of *ags3* and *sph3* expressions

After 48 h static incubation, the fungal cultures were analysed for DNA quantification. Expression of the *ags3* and *sph3* was quantified by real-time polymerase chain reaction (RT- PCR) assay. RNA was extracted by using Allrep fungal DNA/RNA/protein kit. First strand synthesis was performed from total RNA with Quantitec Reverse Transcription kit (Qiagen, Manchester, UK). Forward and reverse primers (Eurofins Genomics, Germany) related to the genes of interest, *ags3* and *sph3* and a reference gene, *sac7* were used (Table 1). A fragment of the gene encoding *ags3* and *sph3* was isolated from genomic DNA using PCR with master mix prepared by adding SYBR Green (Merck, Dorset, UK). RT-PCR was performed using a 7500 RT-PCR System (Fisher Scientific Ltd, Loughborough, UK). Fungal gene expression was normalized to *A. fumigatus sac7* expression. A comparative threshold cycle (Ct) method was used for relative quantification detecting changes in expression of the genes of interest relative to a reference gene.

**Table 1:** Nucleotide sequences of primers used in RT-PCR experiments

Gene	Primer	Sequence (5ʹ > 3ʹ)	Product Size (bp)	References
*sph3*^a^	Forward	GGGCATATGTCCAAGGTCTTTGTGCCTCTCTATGTG	657	Bamford et al. (2015)
	Reverse	GGCTCGAGCTATTTTCCCATCAAATCCACAAACTC		
*ags3*^b^	Forward	CGGCAGTCTCTACCTTGGTC	1700	Maubon et al. (2006)
	Reverse	TCGTTCTTCAGCTTGACAGC		
Rho GTPase activator (*sac7*)	Forward	AGGAGGATGAAAGTAAAGGACCCC	159	Llanos et al. (2015)
	Reverse	AAACCCCACACTTGGCGAC		

They all were provided by Eurofins Genomics, Germany

^a^Encodes a protein belonging to the spherulin-4 family

^b^Encodes cell wall α (1–3) glucan biosynthesis

### Statistical analyses

The SPSS software was used for paired sample T-Test calculation showing data sets that were deemed not significantly different (N.S. > 0.05) and data sets that were significant at different levels: *P ≤ 0.05, **P ≤ 0.01, ***P ≤ 0.001 and ****P ≤ 0.0001 (SEM bars are shown for n = 3).

## Results

### Combination treatment of *A. fumigatus* with triclosan and L-AMB

The MICs for triclosan and L-AMB were calculated as 2 and 1 mg/L, respectively [Supplementary file (S1 and S2)]. In order to investigate triclosan and L-AMB synergistic activity, FICI was adapted as explained in Materials and methods and Table 2 shows that a synergistic activity (FICI ≤ 0.5) between the agents could be achieved if triclosan and L-AMB were applied at doses equal or less than 0.6 and 0.2 mg/L, respectively. The minimum inhibition effect, when they were added simultaneously (triclosan dose at 0.6 mg/L plus L-AMB dose at 0.2 mg/L) and sequentially (L-AMB followed by triclosan) revealed 60.5% and 60% viability, respectively (Table 2). This means the minimum inhibition (MIC50; 50% growth inhibition) was not obtained. Sequential addition of triclosan-L-AMB to *A. fumigatus* cells, first triclosan (0.6 mg/L) followed by L-AMB (0.2 mg/L), showed 48.5% viability (counted as MIC), resulting in FICI = 0.5; hence a synergistic interaction was formed. Therefore, the triclosan and L-AMB MICs were reduced to 30% and 20%, and dropped to 0.6 and 0.2 mg/L, respectively, which present sub-MIC doses in this combination treatment.

**Table 2:** Checkerboard assay analysis of *A. fumigatus* triclosan and L-AMB combination treatment

L-AMB (mg/L)	Triclosan (mg/L)			
	0	0.15	0.3	0.6
Simultaneously combination treatment				
0	100^a^	95.5	93	88
0.05	90.5	70	66	67
0.1	87.7	70	65.5	69
0.2	83	81.5	73	60.5
Sequential combination treatment (L-AMB following by triclosan)				
0	100	96.5	95	95
0.05	92.5	80	77	77
0.1	90.7	70	65.5	65
0.2	90	85	70	60
Sequential combination treatment (triclosan following by L-AMB)				
0	100	90.5	89.5	80
0.05	95	87	70	67
0.1	93	70	65	60
0.2	93	67	55	48.5

^a^Numbers are presented the cells’ viability (%)

**Fig. 1:**
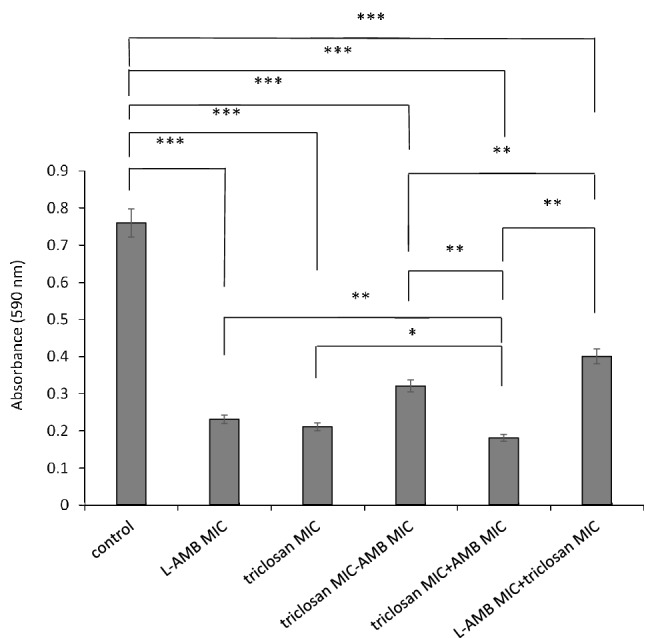
Simultaneous and sequential combination treatments of *A. fumigatus* with triclosan and L-AMB sequential combination treatment (with drug regimens administered one after another) results showed that adding triclosan following with L-AMB is more effective than adding the agents alone, L-AMB following with triclosan, and the simultaneous addition of the agents. (SEM bars represent *P ≤ 0.05, **P ≤ 0.01 and ***P ≤ 0.001)

The effectiveness of triclosan and L-AMB alone and in combination against *A. fumigatus* were analysed (Fig. 1).

To confirm checkerboard assay results, various doses of triclosan (0, 0.15, 0.30, 0.60 and 1.20 mg/L) were combined with L-AMB (0.2 mg/L), sequentially (first triclosan followed by L-AMB) and relevant ODs were analysed using Compusyn software (Table 3).

**Table 3:** CI data for non-constant combination (1:1) of triclosan + L-AMB. CI < 1, = 1, and > 1 indicate synergism, additive effect and antagonism, respectively

Triclosan (mg/L)	L-AMB (mg/L)	CI
0.00	0.20	27.36
0.15	0.20	40.50
0.30	0.20	0.09
0.60	0.20	0.002
1.20	0.20	160.27

Any CI value between 0 and 1 represent synergism, values equal to 1 shows additivity, and anything above 1 shows antagonism. Among 5 combination data points, 2 represent synergy (CI < 1): triclosan (0.6 and 0.3 mg/L) followed by L-AMB (0.2 mg/L). Together, these data support a synergistic activity between triclosan and L-AMB in *A. fumigatus*. This synergism could be through triclosan triggering persister cells and make them vulnerable to L-AMB.

Diminishing of metabolic activities has been reported for different microbes treated with triclosan or L-AMB (Kovács et al. 2017; Westfall et al. 2019). Our study showed that in *A. fumigatus*, a synergistic interaction occurs between triclosan and L-AMB when they were added at their sub-MICs, sequentially (triclosan + L-AMB).

### Structure modifications of biofilm and the viability of conidia in *A. fumigatus* treated with triclosan and L-AMB

**Fig. 2:**
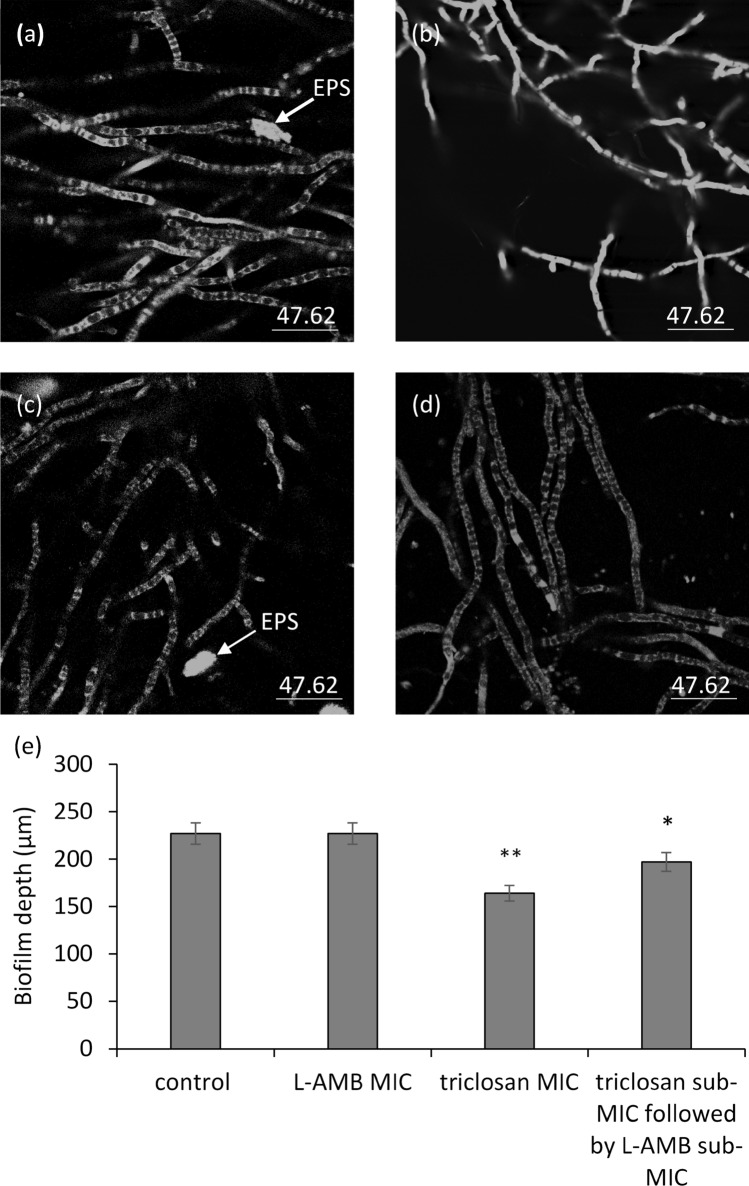
The FUN-1 exposed the morphology of *A. fumigatus* biofilm. *A. fumigatus* conidia were growing on glass surfaces for 36 h (accounted for mature biofilm formation). CLSM images of *A. fumigatus* FUN-1-stained from **a** control; **b** treated with triclosan MIC; **c** treated with L-AMB MIC; and **d** treated with triclosan sub-MIC (0.6 mg/L) following with L-AMB sub-MIC (0.2 mg/L). Bar = 47.62 µm. **e** The graph demonstrates the relevant CLSM microscopy 3D surface plots images of FUN-1-stained *A. fumigatus* at 48 h growth (SEM bars represent *P ≤ 0.05 and **P ≤ 0.01). The arrows show EPS proliferation as regions of bright-green fluorescence without clear edges, as already has been described by Seidler et al. (2008)

In this study, to assess if the lack of viability in the treated samples compared to the untreated control group was because of the lack of cells attachments to a surface or is because of their death by the agents, we used CLSM, PI-based flow cytometry, and CV-based viability assay. As previously shown in Table 2, single treatment with triclosan and L-AMB at their sub-MICs were not effective against *A. fumigatus* and the best result happened when triclosan followed by L-AMB at their sub-MICs. So, CLSM was used to compare triclosan and L-AMB (MICs) single treatments with triclosan-L-AMB (sub-MICs) combination treatment (Fig. 2). According to the confocal imaging, the combination of sub-MIC levels of L-AMB and triclosan against *A. fumigatus* showed a decrease in EPS production and biofilm depth as compared with the L-AMB at its MIC. Triclosan revealed the least biofilm depth and no EPS structure was formed in the samples treated with triclosan MIC. The EPS matrix can encapsulate components released from different compartments (like eDNA, protein, and carbohydrates), which leak out following cell death.

**Fig. 3:**
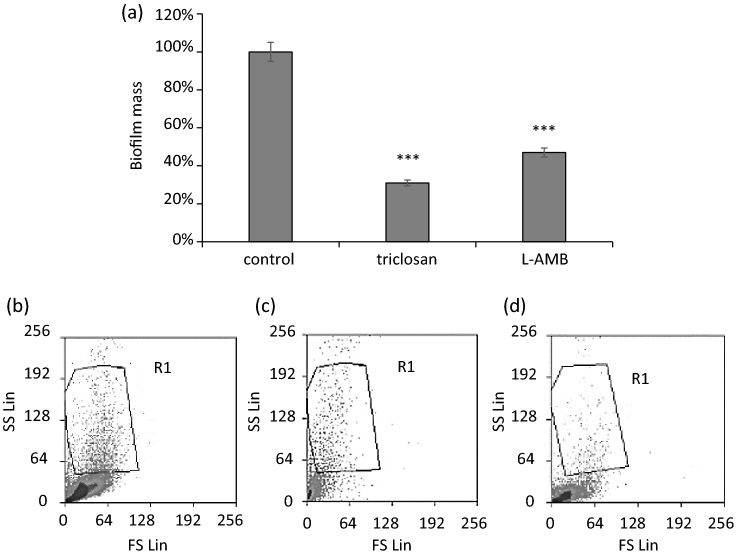
**a** The graph shows indirect measurement of biofilm biomass by adsorption/desorption of CV by using Eq. 2. It shows that the fungus attachment and biofilm formation were less in the agent-treated samples than the untreated control group and that triclosan showed better anti-biofilm effect than L-AMB (SEM bars represent ***P ≤ 0.001). The plots show FSC vs SSC densities. They indicate the effect of triclosan and L-AMB on the conidia cells population. The groups were as follows: **b** untreated control group, **c** triclosan-treated, and **d** L-AMB -treated; R1, the *A. fumigatus* single conidia cells passing through the cytometer isolated based on the cells’ size and granularity

Our findings showed that the total biomass was higher in L-AMB -treated cultures than in samples treated with triclosan (Fig. 3a), whereas the conidial viability of triclosan-treated conidia (~ 75% of the control) was less affected than that of L-AMB -treated test groups (~ 60% of the control) (Fig. 3b–d). CV binds to proteins, polysaccharides and nucleic acids and thus stains the EPS and the microbial cells, so that biofilm viability is not measured by CV (Welch et al. 2012; Kvasničková et al. 2016).

**Fig. 4:**
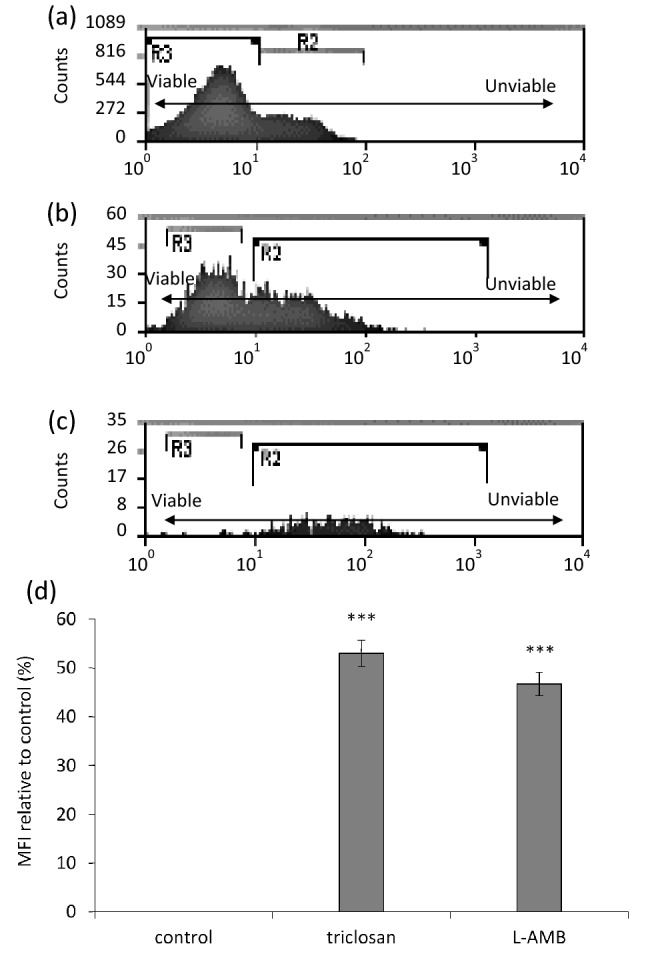
Relative susceptibility to triclosan and L-AMB were measured by forward and side scatter characteristics of the conidial population and by MFI of the PI. a, b and c show flow cytometry measurements of antifungal effects of the agents on *A. fumigatus* conidia suspensions; **a** untreated control group, **b** triclosan-treated, and **c** L-AMB -treated. Approximately 30,000 particles were analysed in each run. Counts, number of particles; R2, conidia passing in pairs through the flow cytometer; R3, conidia passing singly through the flow cytometer. **d** MFI of PI in the antimicrobials-treated conidia in relation to the growth control (standardised to 0% fluorescent). MFI analyses in both FL-1 and FL-2 channels show eDNA increase in agents-treated groups compared with the untreated control group (SEM bars represent ***P ≤ 0.001)

PI-based flow cytometry analysis of the conidial viability (Fig. 4) revealed that the viability was affected the most by triclosan (MFI ~ 52% more than control), and the least by L-AMB (MFI ~ 48% of the control). Persister cells appear to be affected by triclosan but not by L-AMB. So, upon removal of L-AMB (or losing its effectiveness), the persister cells can proceed growing (Fig. 3a).

To sum up, conidial viability was decreased by L-AMB, but viable (attached) conidia were able to form biofilm structures more functional than that in the triclosan-treated samples. As a result, L-AMB had a lower impact on biomass than triclosan (Fig. 3a). In L-AMB -treated cultures, the released eDNA was thus captured in the EPS structure as CLSM results demonstrated (Fig. 2c).

### Expression levels of *A. fumigatus* genes encoding cell wall proteins, α-(1,3)-glucans and GAG

**Fig. 5:**
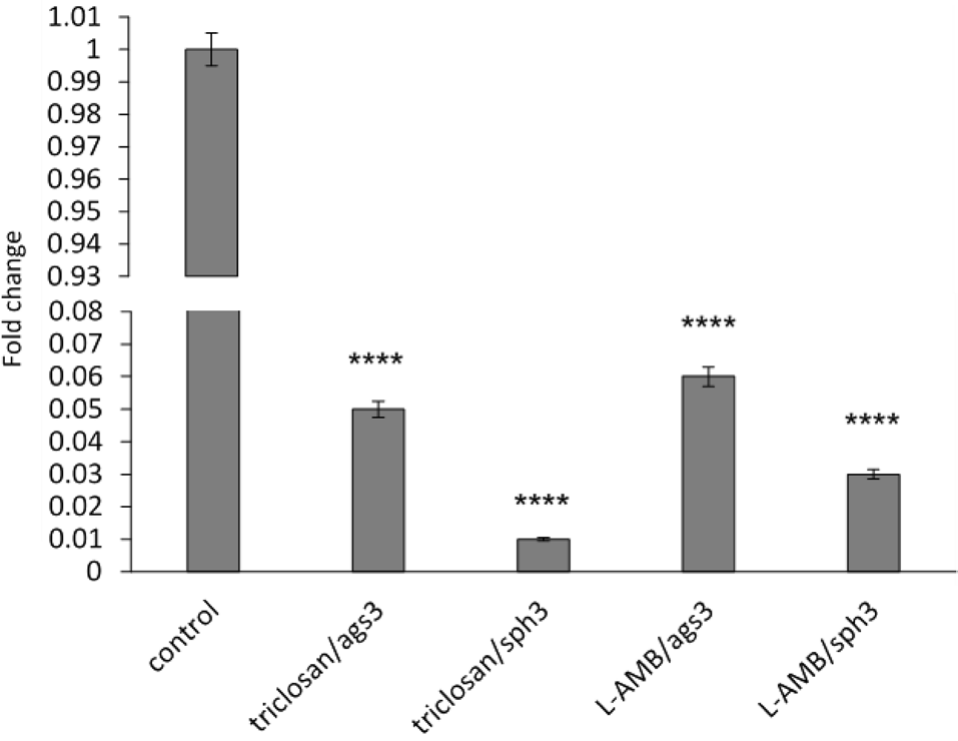
Relative expression of *ags3* and *sph3* in *A. fumigatus* treated with triclosan and L-AMB compared with untreated control. The graph shows that the expression of the genes was down-regulated in triclosan- and L-AMB—treated *A. fumigatus* samples (SEM bars represent ****P ≤ 0.0001)

The sph3 and ags3 genes determine the production of two dominant carbohydrates in *A. fumigatus* conidia cell wall, galactosaminogalactan (GAG) and α-1,3-glucan, respectively. As part of the study of the mechanism of action of triclosan and AMB, the effect of these compounds on the expression of the sph3 and ags3 genes was analyzed. RT-PCR results revealed *ags3* and *sph3* down-regulation in triclosan- and L-AMB -treated conidia (Fig. 5).

## Discussion

Here, we assessed triclosan’s antifungal effect against a filamentous fungus *A. fumigatus*. The antifungal effect was suggested to be through interrupting the quorum sensing signalling system and hence diminishing EPS/biofilm structure in the fungus.

We applied the combination treatment, as a tool to reduce the effective doses for both triclosan and L-AMB. When triclosan and L-AMB were added at their sub-MICs, sequentially (triclosan + L-AMB), a synergistic interaction occurred in *A. fumigatus*. The improvement of the antifungal effect of L-AMB on the cells could be due to triclosan’s action on persister cells, which make them vulnerable to L-AMB effect. Persisters can endure drug concentrations much higher than the MIC and represent survivor cells, which are phenotypic variants of the wild-type cells rather than mutants (Al-Dhaheri and Douglas 2009).

In *A. fumigatus*, L-AMB might not affect the conidial attachment to the substrates, nor the attached cells development proliferation. But, among the cells, most likely non-persister cells or the cells which are not resistant against L-AMB, are killed with L-AMB. The alive cells, though, can grow and form biofilm structure. While in a triclosan-treated sample, no EPS/biofilm structure was formed to capture the released cell components. So, triclosan’s effect might be through its action on the persister cells or through the developmental stage of the cells’ life cycle, where the biofilm structure forms. If the latter concept is the case, a quorum quenching role for triclosan against *A. fumigatus* could be assumed. Whatever the cause, triclosan is a suitable antifungal agent against *A. fumigatus* as it does not induce resistance in the fungus.

The conidia surface of *A. fumigatus* (the rodlet surface) is markedly hydrophobic and is composed of 40% hydrophobic methyl groups (González-Ramírez et al. 2016). In either triclosan- or L-AMB -treated samples, the rodlet structure, if it forms, would be less organized than the rodlet of the untreated strain, which putatively modified the ionic strength of the hydrophobin layer in the triclosan- and L-AMB -treated strains. Absence of the rodlet layer promotes the presence of conidia with more hydrophilicity compared to the conidia of the wild-type strain (Girardin et al. 1999). Therefore, the absence of RodA protein may result in a heterogeneous culture comprising both hydrophobic and hydrophilic conidia. The absence of conidia hydrophobicity may be due to the hydrophilic glycoprotein layer, which is normally secreted as a result of either *ags3* or chitin synthase mutants during vegetative growth. Consequently, conidia become hydrophilic upon treatment with triclosan and L-AMB, because of a rodlet layer masked by a hydrophilic layer or modified by the ionic strength of the rodlet layer. It has also been found that α-(1,3)-glucans interact among themselves and are a reason for the aggregation of swollen conidia without the involvement of any protein (Fontaine et al. 2010). Hence the absence of α-(1,3)-glucans affects biofilm formation through interrupting the microcolony formation stage, which initiates with conidial aggregation.

These findings may help increase understanding of the mechanisms underlying triclosan activity against moulds, which, for example, may provide triclosan as a suitable coating supplement for the abiotic surfaces.

## Supplementary Information

Below is the link to the electronic supplementary material.

Supplementary file1 (DOCX 114 kb)

## Data Availability

All data generated or analysed during this study are included in this published article.
