# Supplementary material for: Antifungal effect of triclosan on Aspergillus fumigatus: quorum quenching role as a single agent and synergy with liposomal amphotericin-B
Source: World J Microbiol Biotechnol. 2022 Jun 20;38(8):142. doi: 10.1007/s11274-022-03325-1 (PMC9206924; doi:10.1007/s11274-022-03325-1)
Supplement: Supplementary file 1 — Supplementary file1 (DOCX 114 kb) [file 11274_2022_3325_MOESM1_ESM.docx]

**Supplementary file**

**Fig. 1** *A. fumigatus* inoculum was cultured and treated with triclosan doses in RPMI1640 medium at 37°C under static condition. Effective dose of triclosan against *A. fumigatus* was determined by resazurine- based viability assay. Its MIC was calculated as 2 mg/L, which inhibits the viability by ~ 72% after 24 h of incubation. **P* ≤ 0.05, **P ≤ 0.01 and ***P ≤ 0.001 (SEM bars are shown for n = 3)

**Fig. 2** *A. fumigatus* inoculum was cultured and treated with L-AMB doses in RPMI-1640 medium at 37°C under static condition. Effective dose of L-AMB against *A. fumigatus* was determined by resazurine- based viability assay. Its MIC was calculated as 1 mg/L, which inhibits the viability by 68% after 24 h of incubation. *P ≤ 0.05 and **P ≤ 0.01 and ***P ≤ 0.001 (SEM bars are shown for n = 3)

**Supplementary file**

**Fig. 1**

**
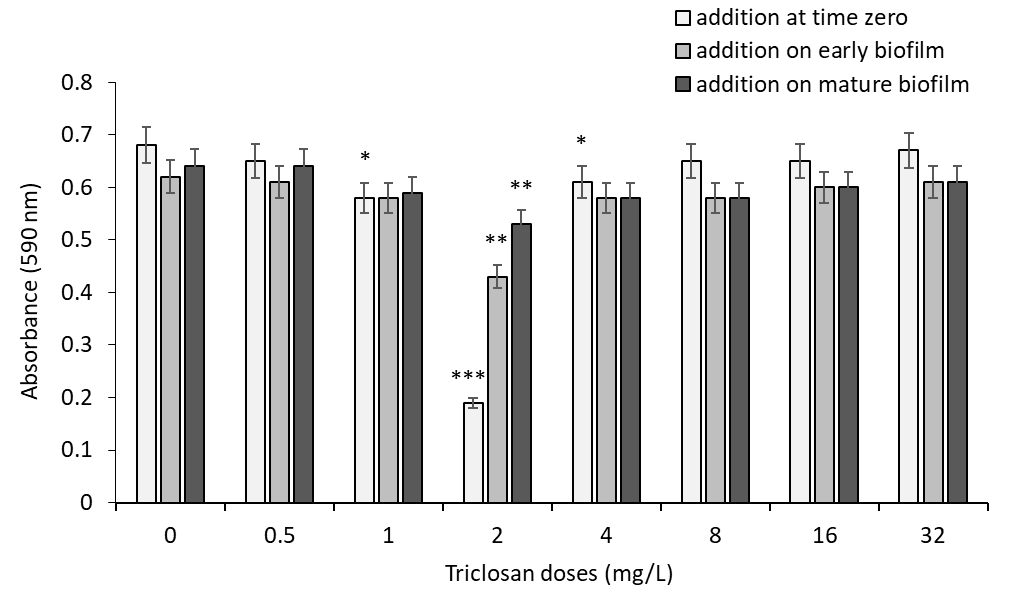
**

**Fig. 2**

**
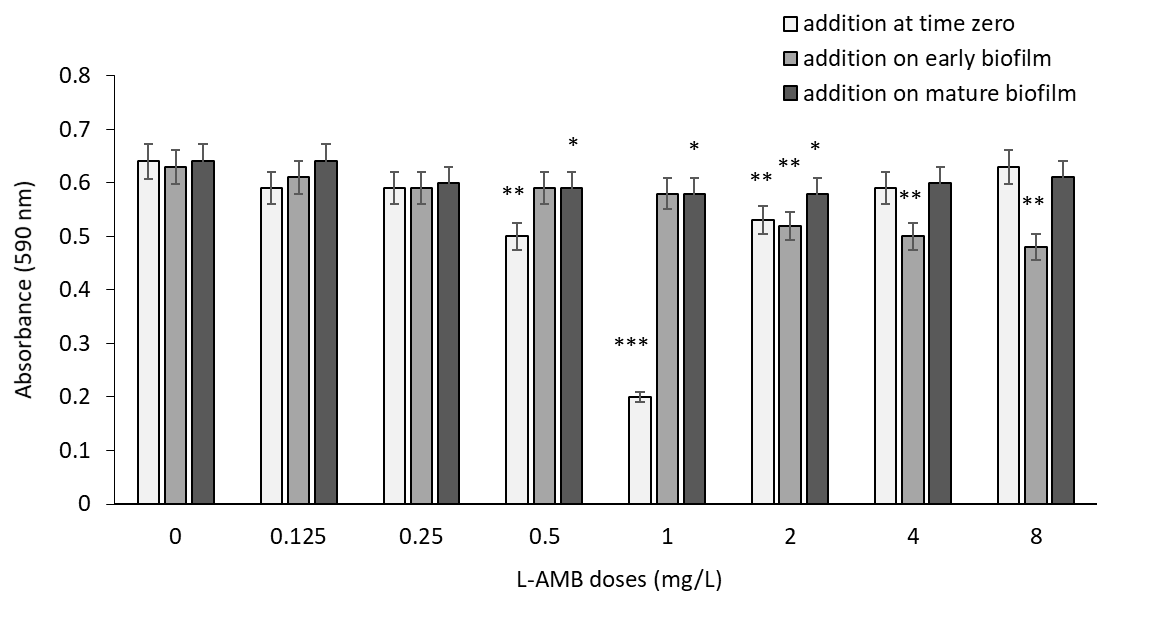
**
